# Determinants of maternal postnatal care utilization in Bangladesh: A machine learning and SHAP-based analysis of BDHS 2022 data

**DOI:** 10.1371/journal.pone.0350188

**Published:** 2026-05-26

**Authors:** Amartay Kumar Dhar, Sharmin Akther, Farhana Akter Bina

**Affiliations:** 1 Department of Statistics and Data Science, Jahangirnagar University, Dhaka, Bangladesh; 2 Faculty of Science and Information Technology, Daffodil International University, Dhaka, Bangladesh; VART Consulting PVT LTD, INDIA

## Abstract

Postnatal care (PNC) plays a crucial role in minimizing maternal and neonatal morbidity and mortality, but the uptake of services in Bangladesh remains below the recommended level. Although logistic regression has been widely used, it may miss complex nonlinear interactions among social, economic, and healthcare factors. This study contributes to the body of knowledge by using machine learning (ML) to identify the most significant determinants of PNC and to enhance prediction accuracy. We compared logistic regression to several ML models, including Random Forest, XGBoost, CatBoost, Support Vector Machine, AdaBoost, and Gradient Boosting, using nationally representative data from the 2022 Bangladesh Demographic and Health Survey (BDHS) with ADASYN oversampling to correct class imbalance. Among all models, Random Forest achieved the highest AUC (0.9050), closely followed by XGBoost (0.9036) and CatBoost (0.9028), all of which substantially outperformed logistic regression (AUC = 0.8470). SHAP analysis of the Random Forest model indicated that delivery place, husband’s occupation, rural residence, wealth index, and media exposure were the most influential predictors of PNC utilization, alongside maternal education, women’s occupation, and age-related factors. The results indicate that ML is more effective than classical procedures for revealing latent patterns and making accurate predictions. Policy implications include encouraging facility-based deliveries, improving maternal education, reducing wealth disparities, and enhancing media coverage of health, particularly among rural and low-income groups. This paper not only identifies key drivers of PNC in Bangladesh but also demonstrates how ML can supplement traditional methods to reinforce maternal health policy and interventions.

## 1. Introduction

The health of mothers and newborns remains one of the most crucial indicators of a health system’s overall performance and development. Postnatal care (PNC), which is a package of services offered to women and their newborn children in the first six weeks of life, is important in cutting down avoidable maternal and neonatal deaths. During this period, they are particularly vulnerable since most of the complications, such as hemorrhage, sepsis, and hypertensive disorders, happen at or immediately after delivery. With timely PNC, it is easy to detect and address the occurrence of complications, provide critical health education, and support the continuum of care to the mother and child [1,2]. World health practices recommended by, but not limited to, the World Health Organization (WHO) include comprehensive PNC, which encompasses newborn screening, maternal mental health assessment, and individualized care, aiming to improve maternal and newborn outcomes [3]. PNC coverage, however, is less than ideal worldwide. The latest Demographic and Health Surveys (DHS) have shown that the average coverage by timely postnatal services provided to mothers is 56 percent, and the newborn coverage is even less [4]. This disparity jeopardizes the achievement of Sustainable Development Goal 3 (SDG 3), which aims to reduce maternal mortality and ensure universal access to quality maternal and newborn care [5].

In Bangladesh, maternal health continues to be a public health priority. The maternal mortality ratio stands at 196 per 100,000 live births, with hemorrhage (31%) and eclampsia (23%) as the leading causes of death. According to the BDHS 2022, maternal PNC utilization has increased to 76.6%, up from 63.6% in 2018 [6]. However, newborn PNC coverage remains alarmingly low, and significant disparities persist across wealth quintiles, rural-urban divides, and education levels [7,8]. These persistent inequities highlight the need for evidence-based interventions to enhance equitable postnatal care coverage in Bangladesh [9,10]. Although analyses involving regression, and especially logistic regression, have been extensively used in Bangladesh to determine PNC determinants [9]. Regression models assume linear relationships between predictors and outcomes, and do not well induce complex interactions. They have also been found to perform poorly with disproportional health information, like the relatively low percentage of mothers who obtain PNC. In comparison, machine learning methods can flexibly express nonlinear relationships, work with high-dimensional categorical data, and find subtle patterns that are missed with other methods. [10,11]. Studies in other developing countries, such as India and Ethiopia, have demonstrated the potential of ML techniques to identify key predictors of PNC [12,13]. Furthermore, explainable AI procedures like SHAP deliver clear explanations about these models, so the analytical advantages are converted into policy intelligence. It is against this background that our study uses a machine learning framework on the BDHS 2022 data to build on and extend conventional methods, providing a better predictive capacity as well as further insight into the factors that drive maternal PNC in Bangladesh.

Here, we use a BDHS 2022 dataset and explore ML models, namely Logistic Regression, Random Forest, Support Vector Machine (SVM), AdaBoost, Gradient Boosting, XGBoost, and CatBoost, to determine factors influencing the utilization of postnatal care among the selected mothers. Moreover, we use SHapley Additive exPlanations (SHAP) to improve the interpretability of a model, which will help to determine the impact of an individual set of features on maternal PNC predictions [14].

This study aims to: (i) identify the key socio-demographic and healthcare determinants of maternal PNC in Bangladesh using BDHS 2022 data, and (ii) leverage ML and SHAP-based interpretability to provide actionable insights for policy and program design. This research will facilitate the use of targeted interventions and evidence-based decision-making through the integration of state-of-the-art machine learning models with the explainable AI approach, thereby ultimately helping the SDG 3 agenda for maternal and newborn health.

## 2. Methods and materials

### 2.1 Data source

**Fig 1:**
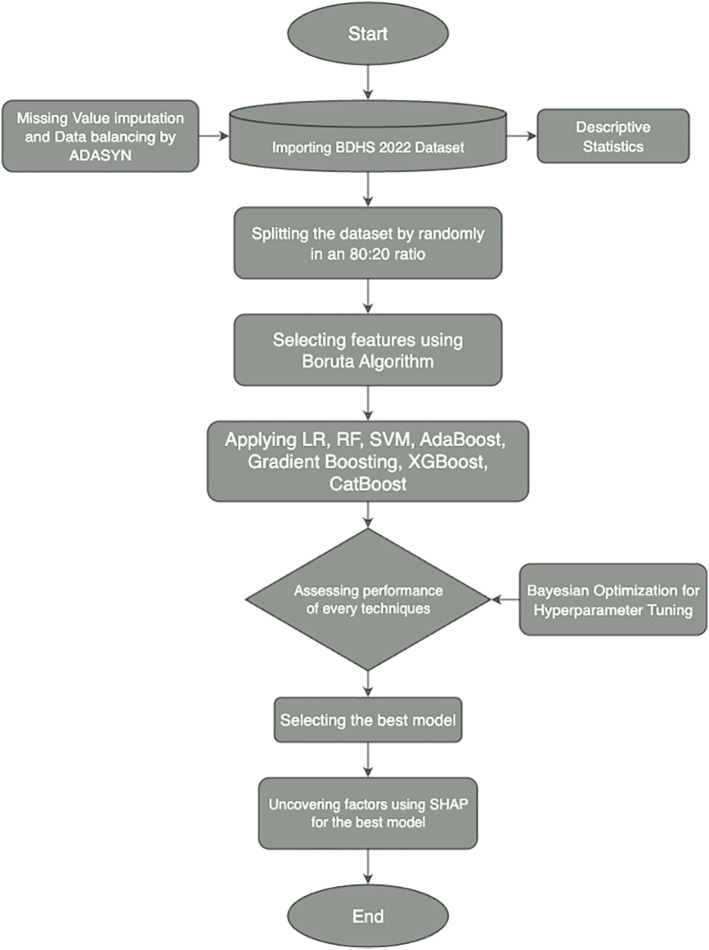
Participant flow diagram showing the derivation of the final analytical sample from the BDHS 2022 dataset.

This cross-sectional study was conducted to determine the risk factors of Maternal Postnatal Care in Bangladesh using a secondary database from the 9th national survey (BDHS-2022). In the first identification, a sample of 675 listing units was selected using PPS sampling: 237 in the urban environment and 438 in the rural environment. To replace a household selection frame in the second stage, a full family list was created in each of the enumeration units sampled in the first stage of the selection. Then, systematic sampling was employed, and 30 households in each listing unit were chosen in the second phase. After the sampling process, only women aged 15–49 who had a birth in the 3 years preceding the survey were included, because PNC is relevant only to women who have recently given birth. A total of 2,092 women who had a live birth in the three years before the BDHS 2022 were eligible for PNC assessment among the 30,078 women interviewed in the survey. Records with key covariates missing were then excluded: 17 records were missing husband’s occupation, 4 records were missing BMI, and 209 records were missing household size, for a total of 226 exclusions. After applying all eligibility and data quality filters, a final analytical sample of 1,866 women was obtained. Fig 1 shows the derivation of the final sample.

In line with the DHS analytical guidelines, all the analyses were done with the BDHS complex survey design that includes sampling weights, stratification, and clustering to generate nationally representative estimates and valid standard errors. All descriptive and inferential analyses were done with the sampling weight to account for unequal selection probabilities [6].

**Fig 2:**
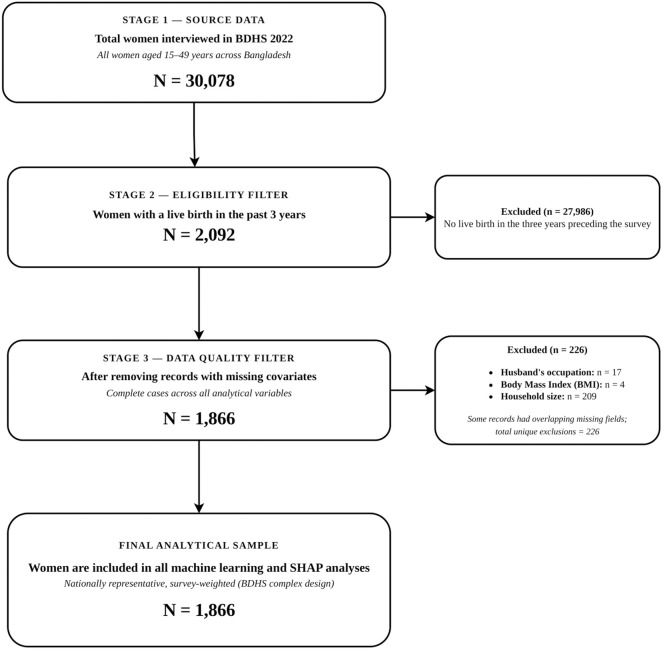
Machine learning workflow for analyzing maternal postnatal care determinants using BDHS 2022 data.

This study utilized data from the Bangladesh Demographic and Health Survey (BDHS) 2022 to identify determinants of maternal postnatal care utilization. A structured analytical framework was adopted, integrating classical statistical methods with advanced machine learning algorithms. Fig 2 presents the comprehensive workflow of the study. To address class imbalance in PNC utilization, we applied ADASYN (Adaptive Synthetic Sampling) only to the training folds, ensuring that synthetic data were never introduced into the test set. This prevents information leakage and preserves the integrity of model evaluation. We also conducted sensitivity checks with class-weighted loss functions to confirm that results were not dependent solely on oversampling. Feature selection was conducted using the Boruta algorithm to identify the most relevant predictors. A range of machine learning models, including Logistic Regression, Random Forest, Support Vector Machine, AdaBoost, Gradient Boosting, XGBoost, and CatBoost, were applied, and their performance was rigorously assessed using ten-fold cross-validation and standard evaluation metrics such as accuracy, sensitivity, specificity, F1 score, precision, and Cohen’s kappa. Bayesian Optimization was used to fine-tune model hyperparameters. The best-performing model was selected and interpreted using SHAP (SHapley Additive exPlanations) values to uncover key factors influencing maternal postnatal care outcomes, ensuring both robust predictions and interpretability.

### 2.2 Outcome variable

The outcome variable of this study is maternal postnatal care (PNC) utilization. Based on the BDHS 2022 data, women who received a postnatal health check within 2 days (48 hours) of delivery were coded as ‘Yes’ (PNC = 1), consistent with the WHO recommendation for timely postnatal care. All remaining women who either did not receive a postnatal check or received it beyond 2 days after delivery were coded as ‘No’ (PNC = 0).

### 2.3 Covariates

The independent variables encompass a wide range of maternal, child, household, and community-level characteristics. Regional factors include Division (Barisal, Chittagong, Dhaka, Khulna, Mymensingh, Rajshahi, Rangpur, Sylhet) and Residence (Urban, Rural). Maternal characteristics include Mother’s Education (No education, Primary, Secondary, Higher), Mother’s Working Status (No, Yes), Body Mass Index (Underweight, Normal Weight, Overweight, Obese), and Women’s Age Group (15–19, 20–24, 25–29, 30–34, 35+). Reproductive health variables include Children Ever Born (1 child, 2 children, 3 or more), Marriage Age Group (<16, 16–21, 22 or more), First Birth Age Group (<16, 16–21, 22 or more), and Mother Age Group (<18, 18–25, 26–35, 36 or more). Health service-related variables include Place of Delivery (Home, Others), Type of Toilet Facility (Hygienic, Unhygienic), Distance to Health Facility (Big problem, Not a big problem), and Media Coverage (No, Yes). Household factors include Wealth Index (Poorest, Poorer, Middle, Richer, Richest) and Household Size (<3, 4–6, 7+). Religious affiliation is categorized as Muslim and Non-Muslim. Occupational status is included for both the husband (Unemployed, Service, Farmer/Others, Others) and the woman (Housewife, Others). Child mortality experience is reflected by the number of children who died (No child died, 1 child died, 2 or more died). All variables were recoded before statistical analysis to ensure clarity and consistency in interpretation.

**Table:** 

Factors	Description	Factor Types	Class Levels
Division	Administrative region of residence	Categorical	Barisal, Chittagong, Dhaka, Khulna, Mymensingh, Rajshahi, Rangpur, Sylhet
Residence	Type of locality	Categorical	Urban, Rural
Mother Education	Highest educational attainment of the mother	Categorical	No education, Primary, Secondary, Higher
Wealth Index	Household economic status	Categorical	Poorest, Poorer, Middle, Richer, Richest
Distance to Health Facility	Perceived problem with accessing health facility	Categorical	Big problem, Not a big problem
Mother Working Status	Whether the mother is employed	Categorical	Yes, No
BMI	Body Mass Index of the mother	Categorical	Underweight, Normal Weight, Overweight, Obese
Type of Toilet	Type of sanitation facility used	Categorical	Hygienic, Unhygienic
Delivery Place	Location of childbirth	Categorical	Home, Others
Religion	Religious affiliation	Categorical	Muslim, Non-Muslim
Children Ever Born	Total number of children ever born	Categorical	1 child, 2 children, 3 or more children
Media Coverage	Exposure to mass media	Categorical	Yes, No
Husband Occupation	Main occupation of the husband	Categorical	Farmer or household, Service holder, Unemployed, Others
Women Occupation	Main occupation of the mother	Categorical	Housewife, Others
Women Age Group	Age group of the mother	Categorical	15–19, 20–24, 25–29, 30–34, 35+
Children Died	Number of children died	Categorical	No child died, 1 child died, 2 or more children died
Marriage Age Group	Age at first marriage	Categorical	<16, 16–21, 22 or more
First Birth Age Group	Age when the mother had her first child	Categorical	<16, 16–21, 22 or more
Household Size	Number of people in the household	Categorical	<3, 4–6, 7 or more

### 2.4 Chi-square test

We initially conducted chi-square tests to examine bivariate associations between maternal PNC utilization and each covariate. To address multiple comparisons, we applied the Benjamini–Hochberg procedure to control the false discovery rate (FDR) at the 5% significance level. This adjustment reduces the likelihood of spurious significance when conducting multiple hypothesis tests. Additionally, our primary inferential focus was on variables retained through the Boruta feature selection and SHAP-based model interpretation, ensuring that findings were not solely dependent on unadjusted bivariate associations.

### 2.5 Feature selection method

#### 2.5.1 Boruta algorithm.

We used the Boruta algorithm to estimate feature importance for potential determinants of maternal PNC, ultimately identifying 15 significant variables. This methodological approach enhances the reliability and generalizability of our results and directly advances understanding by clarifying which factors significantly affect maternal PNC.

### 2.6 Traditional model

#### 2.6.1 Logistic regression.

Logistic regression is a statistical model for predicting the outcome of a binary random variable. It approximates the likelihood of a certain type or occurrence, which can be used in many contexts of machine learning, medicine, and the social sciences, in classifications [15].

### 2.7 Machine learning models

#### 2.7.1 Random forest.

Random forests (RFs) are ensemble learning algorithms that combine decision trees and are applicable to regression and classification. It uses a combination of bagging and random subspace methods to increase the accuracy of predicting and reducing the variance, as well as making the model very stable because it averages multiple decision trees [16].

#### 2.7.2 SVM.

SVM Support vector machine is a widely used classification tool, which is used as a penalized minimization problem centered on the expectation about the hinge loss functional view of the true underlying measure for the empirical measure generated by a machine [17].

#### 2.7.3 AdaBoost.

AdaBoost is a machine learning algorithm that belongs to the booster family and implies distribution and input feature space identical to training and testing data sets. It improves the performance of the poor classifiers by giving weights to the instances that are being misclassified during training time. It has a tendency, however, to not work well in practice, and so an extension of AdaBoost, TrAdaBoost, was developed to adapt it to the situation in which data distributions vary [18].

#### 2.7.4 Gradient boosting.

Gradient boosting is an additive expansion algorithm, used to approximate a function by training a sequence of models one at a time. It emphasizes the minimization of the loss to increase the predictive performance in the use of machine learning because it trains models with pseudo-residuals of the preceding iterations [19].

#### 2.7.5 XGBoost.

XGBoost is an optimized implementation of gradient boosting that improves the capability of machine learning algorithms. It adds a regularization term to the loss function, and the latter effectively bounds model complexity, enhances the generalization capacity, and also prevents the problem of overfitting [20].

#### 2.7.6 CatBoost.

CatBoost refers to a machine learning algorithm created to solve classification and prediction problems, especially in credit rating. It focuses on tuning of parameters and selection of features; thus, it is an effective tool in predictive analytics, making accurate predictions of classification [21].

### 2.8 Hyperparameter optimization

#### 2.8.1 Bayesian optimization.

Bayesian Optimization serves as a successful optimization algorithm that is used to optimize in gradients of the non-analytical and expensive to evaluate blackbox functions [22]. It is particularly applied in an attempt to optimize hyperparameters of machine learning models. Bayesian Optimization estimates the objective function with Gaussian Processes, thereby constructing a probability model over the objective, and subsequent query points are determined through the optimization of a utility. It is a good balance of exploration and exploitation, and less of them is demanded, compared to the grid or random search [23].

### 2.9 Statistical analysis

A highly structured analytical procedure was used in this research paper, with classical statistical analysis and modern machine learning algorithms used to uncover factors influencing maternal postnatal care. The respondents’ demographic data have been presented using descriptive statistics. Subsequently, the associations between PNC outcomes and a set of sociodemographic and anthropometric factors were evaluated using the Chi-square test. Feature selection was performed using the Boruta algorithm within the training set only. An 80:20 ratio was used to separate the data into a training and a testing set. This division was selected because it provides sufficient training data (n = 1,492) for the models to learn complex patterns and still maintains a sufficient held-out test set (n = 374) to assess model performance without bias. The 80:20 ratio has been widely used in the machine learning literature involving moderate-sized sample sizes and is the ratio that strikes a balance between the model’s learning capacity and the reliability of its evaluation [24]. Specifically, Boruta was implemented inside each resampling fold during cross-validation, ensuring that the test data were never used in the feature selection process. This approach safeguards against optimistic bias and preserves the integrity of model evaluation. Seven machine learning algorithms were used during the modeling step: Logistic Regression, Random Forest, Support Vector Machine (SVM), AdaBoost, Gradient Boosting, and XGBoost. Bayesian Optimization was used to optimize hyperparameters and improve the model’s generalizability. Model performance was also assessed with a complete list of classification measures, such as Accuracy, Cohen’s Kappa, Area Under the Curve (AUC), Precision, Recall, and F1-Score. All of these measures were chosen because they are effective at handling imbalanced health datasets and provide balanced insights into classifier behavior [25–27]. It should also be noted that the precision, recall, and F1 scores for Class 0 and Class 1 reflect the inherent class imbalance in the original data, where 77.5% of observations were Class 0. Even though the ADASYN oversampling was applied only to the training folds to correct the imbalance, the test set was not adjusted to restore its original distribution to create a realistic performance evaluation. Consequently, some models demonstrated stronger results for the majority class, a well-known feature of unbalanced health data. Also, ROC curves were employed in measuring the discrimination of the classifier. The most successful model was also explained with the help of SHAP (SHapley Additive exPlanations) values to make the contribution of features transparent. We conducted all of the analyses in Stata (version 14), R (version 4.1.1), and Python (3.13.5).

### 2.10 Ethics approval and consent to participate

This study is a secondary analysis of publicly available, fully anonymized data from the 2022 Bangladesh Demographic and Health Survey (BDHS). The original BDHS 2022 survey protocol received ethical approval from the ICF Institutional Review Board (IRB) and the Bangladesh Medical Research Council (BMRC) National Research Ethics Committee. Informed consent was obtained from all participants (or their parents/guardians for minors) during the primary data collection. As this is a secondary analysis of de-identified public data, no additional ethics approval or consent was required for the present study.

## 3 Results

Table 1 gives the background characteristics of the distribution of maternal postnatal care utilization. The uptake of PNC was greater among urban mothers (25.98%) than among rural mothers (20.81%; p = 0.022) and among women in the richest wealth quintile (27.25%) than among women in the poorest wealth quintile (15.94%; p = 0.016). The use of facilities-based delivery was closely linked with increased PNC use (26.58%) compared with home births (18.28%; p = 0.002). The occupation of the husband was also important (p = 0.014), mothers whose husbands were unemployed exhibited the highest PNC uptake (28.95%), even though service holders (25.96%). The occupation of women was also important (p = 0.011), and the highest PNC coverage was reported among housewives (24.19%) compared with women in other occupations (17.67). A statistical difference was found in geographic variation (p = 0.024), with Dhaka division showing the highest PNC uptake (31.51) and Mymensingh division the lowest (19.55). The strongest associates of PNC utilization were wealth index, place of delivery, husband’s occupation, residence, and division. Other variables such as maternal education, religion, media coverage, BMI, distance to the health facility, number of children born in the family, child mortality, age at marriage, mothers’ working status, and household size were not statistically significant for PNC utilization.

**Table 1:** Descriptive statistics: Frequencies, percentages, and p-values.

Variable	Category	Total N (%)	PNC		P value
			No N (%)	Yes N (%)	
**Residence**	Urban	612 (32.88)	453 (74.02)	159 (25.98)	**0.022**
	Rural	1,254 (67.12)	993 (79.19)	261 (20.81)	
**Mother Education**	No education	98 (5.26)	78 (79.59)	20 (20.41)	0.463
	Primary	456 (24.44)	364 (79.82)	92 (20.18)	
	Secondary	966 (51.77)	746 (77.23)	220 (22.77)	
	Higher	346 (18.54)	258 (74.57)	88 (25.43)	
**Women Age Group**	15-19	235 (12.59)	175 (74.47)	60 (25.53)	0.845
	20-24	561 (30.06)	436 (77.72)	125 (22.28)	
	25-29	553 (29.63)	436 (78.84)	117 (21.16)	
	30-34	341 (18.27)	261 (76.54)	80 (23.46)	
	35+	176 (9.43)	138 (78.41)	38 (21.59)	
**Women Occupation**	Housewife	1,385 (74.22)	1,050 (75.81)	335 (24.19)	**0.011**
	Others	481 (25.78)	396 (82.33)	85 (17.67)	
**Husband Occupation**	Unemployed	38 (2.04)	27 (71.05)	11 (28.95)	**0.014**
	Service	913 (48.93)	676 (74.04)	237 (25.96)	
	Farmer/Others	351 (18.81)	281 (80.06)	70 (19.94)	
	Others	564 (30.23)	462 (81.91)	102 (18.09)	
**Media Coverage**	No	845 (45.28)	661 (78.22)	184 (21.78)	0.271
	Yes	1,021 (54.72)	785 (76.88)	236 (23.12)	
**Delivery Place**	Home	914 (48.98)	747 (81.72)	167 (18.28)	**0.002**
	Others	952 (51.02)	699 (73.42)	253 (26.58)	
**Children Ever Born**	1 child	613 (32.85)	463 (75.53)	150 (24.47)	0.584
	2 children	643 (34.46)	496 (77.14)	147 (22.86)	
	3 or more	610 (32.69)	487 (79.84)	123 (20.16)	
**Religion**	Muslim	1,719 (92.12)	1,333 (77.54)	386 (22.46)	0.709
	Non-Muslim	147 (7.88)	113 (76.87)	34 (23.13)	
**Wealth Index**	Poorest	414 (22.19)	348 (84.06)	66 (15.94)	**0.016**
	Poorer	382 (20.47)	292 (76.44)	90 (23.56)	
	Middle	359 (19.24)	276 (76.88)	83 (23.12)	
	Richer	366 (19.61)	279 (76.23)	87 (23.77)	
	Richest	345 (18.49)	251 (72.75)	94 (27.25)	
**Type of Toilet**	Hygienic	905 (48.50)	687 (75.91)	218 (24.09)	0.180
	Unhygienic	961 (51.50)	759 (78.98)	202 (21.02)	
**BMI**	Underweight	124 (6.65)	106 (85.48)	18 (14.52)	0.067
	Normal Weight	541 (28.99)	417 (77.08)	124 (22.92)	
	Overweight	220 (11.79)	159 (72.27)	61 (27.73)	
	Obese	981 (52.57)	764 (77.88)	217 (22.12)	
**Distance to Health Facility**	Big problem	854 (45.77)	662 (77.52)	192 (22.48)	0.926
	Not a big problem	1,012 (54.23)	784 (77.47)	228 (22.53)	
**Children Died**	No child died	1,765 (94.59)	1,373 (77.76)	392 (22.24)	0.111
	1 child died	97 (5.20)	71 (73.20)	26 (26.80)	
	2 or more died	4 (0.21)	2 (50.00)	2 (50.00)	
**Mother Age Group**	<18	49 (2.63)	37 (75.51)	12 (24.49)	0.739
	18-25	864 (46.30)	662 (76.62)	202 (23.38)	
	26-35	831 (44.53)	652 (78.46)	179 (21.54)	
	36 or more	122 (6.54)	95 (77.87)	27 (22.13)	
**Marriage Age Group**	<16	585 (31.35)	452 (77.26)	133 (22.74)	0.955
	16-21	1,098 (58.84)	855 (77.87)	243 (22.13)	
	22 or more	183 (9.81)	139 (75.96)	44 (24.04)	
**First Birth Age Group**	<16	196 (10.50)	150 (76.53)	46 (23.47)	0.065
	16-21	1,296 (69.45)	1,021 (78.78)	275 (21.22)	
	22 or more	374 (20.04)	275 (73.53)	99 (26.47)	
**Mother Working Status**	No	1,422 (76.20)	1,088 (76.51)	334 (23.49)	0.167
	Yes	444 (23.80)	358 (80.63)	86 (19.37)	
**Household Size**	<3	19 (1.02)	13 (68.42)	6 (31.58)	0.733
	4-6	1,224 (65.60)	955 (78.02)	269 (21.98)	
	7 or more	623 (33.39)	478 (76.73)	145 (23.27)	
**Division**	Barisal	222 (11.90)	173 (77.93)	49 (22.07)	**0.024**
	Chittagong	429 (22.99)	338 (78.79)	91 (21.21)	
	Dhaka	219 (11.74)	150 (68.49)	69 (31.51)	
	Khulna	175 (9.38)	132 (75.43)	43 (24.57)	
	Mymensingh	266 (14.26)	214 (80.45)	52 (19.55)	
	Rajshahi	167 (8.95)	131 (78.44)	36 (21.56)	
	Rangpur	166 (8.90)	131 (78.92)	35 (21.08)	
	Sylhet	222 (11.90)	177 (79.73)	45 (20.27)	

**Fig 3:**
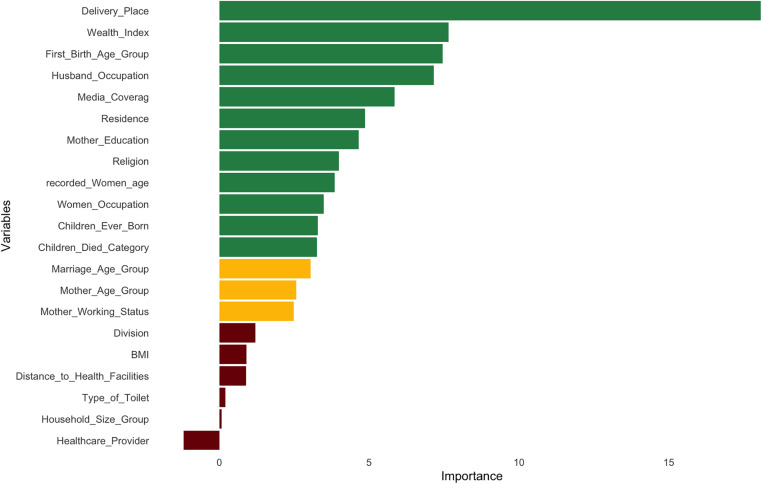
Feature selection using the Boruta algorithm.

Fig 3 illustrates the feature importance rankings derived from the Boruta algorithm for predicting maternal PNC utilization. Variables are color-coded by their confirmation status: green bars indicate confirmed important variables, yellow bars denote tentative variables, and red bars represent rejected variables. Among the 12 confirmed predictors, Delivery Place emerged as the most important variable by a substantial margin (importance ≈ 17), followed by Wealth Index, First Birth Age Group, and Husband Occupation with broadly similar importance scores (approximately 7–8). Media Coverage, Residence, Mother Education, Religion, Recorded Women Age, Women Occupation, Children Ever Born, and Children Died categories were also confirmed as significant, with progressively declining importance scores. Three variables Marriage Age Group, Mother Age Group, and Mother Working Status were classified as tentative, indicating uncertain importance. The remaining six variables, Division, BMI, Distance to Health Facilities, Type of Toilet, Household Size Group, and Healthcare Provider, were rejected, with Healthcare Provider receiving a negative importance score. The 12 confirmed variables were carried forward into subsequent machine learning model development.

**Fig 4:**
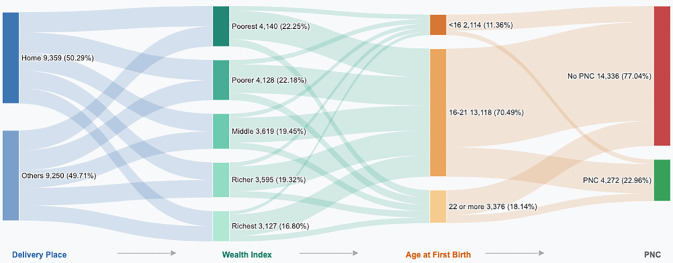
Association among delivery place, first birth age group, wealth index, and husband occupation with PNC.

Fig 4 presents a Sankey diagram illustrating the associations among Delivery Place, Wealth Index, Age at First Birth, and PNC utilization. Regarding delivery place, births were nearly evenly split between home (50.29%) and other facility-based settings (49.71%). These women were distributed across five wealth quintiles, with the Poorest (22.25%) and Poorer (22.18%) groups together comprising the largest share, followed by Middle (19.45%), Richer (19.32%), and Richest (16.80%). In terms of age at first birth, the majority of women gave birth for the first time between ages 16 and 21 (70.49%), while smaller proportions gave birth before age 16 (11.36%) or at age 22 or older (18.14%). Ultimately, the diagram shows that the majority of women (77.04%) did not receive PNC, while only 22.96% did. The flow patterns reveal that lower wealth quintiles and home deliveries predominantly channel toward non-utilization of PNC, while facility-based deliveries and higher wealth groups show relatively stronger flows toward PNC receipt, underscoring the interplay of socioeconomic status, delivery setting, and maternal age at first birth in determining postnatal care uptake.

**Fig 5:**
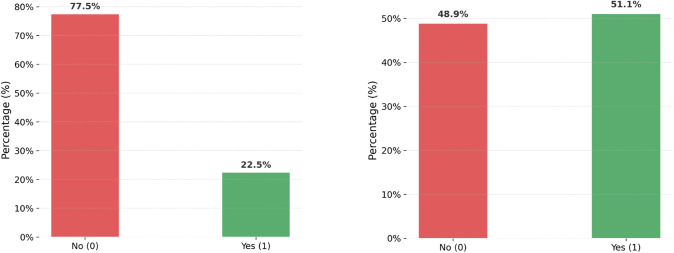
Transformation of class distribution before and after ADASYN application for data balancing.

Fig 5 shows the class distribution before the application of ADASYN. The data is unbalanced, with 77.5% of the samples falling into the “No (0)” class and just 22.5% into the “Yes (1)” class. Machine learning models may perform poorly as a result of this imbalance, frequently becoming biased in favor of the majority class. With 49.5% of the classes being “No (0)” and 50.5% being “Yes (1),” the second image displays the class distribution following the application of ADASYN, which has almost balanced the classes. By ensuring that all classes are equally represented in the model during training, this balancing can improve classification performance, particularly for the minority class.

Table 2 contains a detailed comparison of seven machine learning classifiers according to various performance measures such as accuracy, Cohen’s Kappa, AUC, precision, recall, and F1 score in two categories (0 = No PNC, 1 = PNC received). CatBoost and XGBoost performed better than other models, achieving the highest AUC scores (0.921 and 0.9161, respectively), indicating better discrimination. Random Forest also gave good results, with an AUC of 0.9147 and the highest accuracy (0.8585) and Cohen’s Kappa (0.7154) of all the models, indicating balanced performance across both classes.

**Table 2:** The predictive models’ performance for PNC.

Model	Accuracy	Cohen Kappa	Precision		Recall		F1 Score	
			Class 0	Class 1	Class 0	Class 1	Class 0	Class 1
**Logistic Regression**	0.8226	0.6516	0.7264	0.9941	0.9955	0.6709	0.8399	0.8011
**Random Forest**	0.8585	0.7154	0.8573	0.8595	0.8366	0.8777	0.8468	0.8685
**SVM**	0.8247	0.6559	0.7273	1.0000	1.0000	0.6709	0.8422	0.8030
**AdaBoost**	0.8112	0.6275	0.7306	0.9343	0.9444	0.6942	0.8239	0.7966
**Gradient Boosting**	0.8283	0.6586	0.7714	0.8965	0.8993	0.7660	0.8304	0.8261
**XGBoost**	0.8465	0.6911	0.8448	0.8478	0.8227	0.8673	0.8336	0.8575
**CatBoost**	0.8361	0.6719	0.8087	0.8627	0.8507	0.8233	0.8292	0.8425

SVM had moderate overall accuracy (0.8247), perfect recall for Class 0 (1.000) and perfect precision for Class 1 (1.000), and a low recall for Class 1 (0.6709), suggesting it is highly biased toward the majority class. Class 1 was extremely underpredicted in the model, with the appropriateness of the key variables missing about 33% of actual PNC cases. This performance can be attributed to SVM’s established sensitivity to class imbalance; thus, this approach is not the most appropriate for this classification problem, as opposed to ensemble approaches like Random Forest, XGBoost, and CatBoost.. AdaBoost had the lowest accuracy (0.8112), and Cohen’s Kappa (0.6275), and Gradient boosting had the lowest recall of class 1 (0.7660), although the overall accuracy was moderate (0.8283). CatBoost has shown good, balanced results across the two classes, with a precision of 0.8087 and 0.8627, and a recall of 0.8507 and 0.8233 for classes 0 and 1, respectively. Based on overall performance, Random Forest achieved the highest accuracy (0.8585), Cohen’s Kappa (0.7154) making it the best-performing model for predicting maternal PNC utilization.

**Fig 6:**
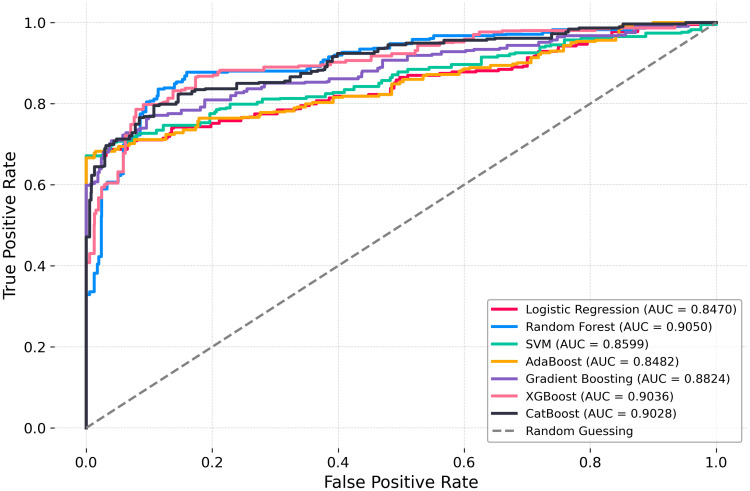
ROC curves for PNC prediction models.

The Receiver Operating Characteristic (ROC) curves for all 7 machine learning classifiers tested in this paper are shown in Fig 6, along with the random guessing baseline (dashed diagonal line). The main measure of the discriminative power of each model is the area under the receiver operating characteristic (ROC) curve (AUC). Random Forest also had the highest AUC (0.9050), closely followed by XGBoost (0.9036) and CatBoost (0.9028). The three ensemble models showed good, similar classification results. Gradient Boosting achieved an AUC of 0.8824, whereas SVM (0.8599) and AdaBoost (0.8482) showed moderate discrimination. The lowest AUC was obtained with Logistic Regression (0.8470), but it was still significantly higher than the random-guessing level. In general, all seven models proved to be reasonably discriminative with an AUC of more than 0.84, and the three models of ensemble boosting and bagging, that is, Random Forest, XGBoost, and CatBoost, were unambiguously superior in comparison with the rest of the classifiers, which supports their appropriateness in predicting maternal PNC utilization in Bangladesh.

**Fig 7:**
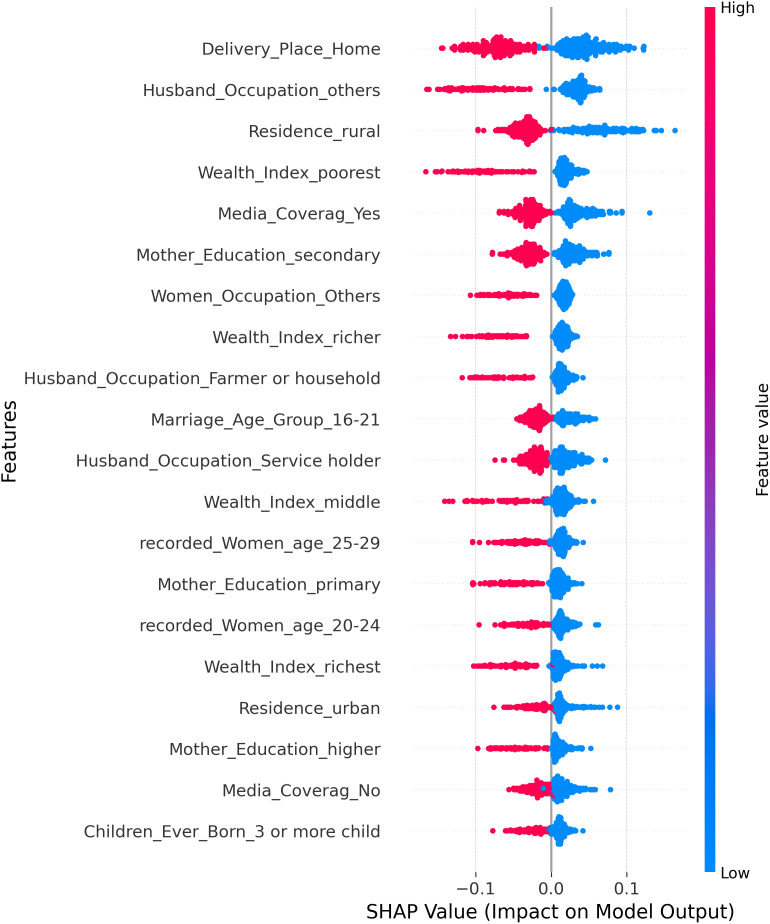
Global SHAP summary plot (beeswarm) for the random forest model in predicting maternal PNC.

The global SHAP summary beeswarm plot for the Random Forest model is shown in Fig 7 and shows how the top 20 features affect the prediction of maternal PNC. The dots represent each observation; the position along the x-axis indicates the size and direction of the SHAP value, and the color of the dots indicates the feature value: red for high values and blue for low values. The features are ordered by decreasing mean absolute SHAP value; the Delivery Place Home feature is the most significant predictor, with high values (red, meaning home delivery) associated with strongly negative SHAP values, indicating a decreased likelihood of receiving PNC. On the same note, Husband Occupation Others and Residence Rural indicate a positive correlation, with higher scores on these features advancing the predictions towards non-utilization of PNC. Wealth Index, Poorest, and Media coverage are also ranked among the best predictors, and the negative contributions of SHAP have consistently been associated with low wealth and a lack of media exposure. The positive SHAP values on the other features, which include Residence Urban, Wealth Index Richest, and Mother Education Higher, on the other hand, show that they increase the likelihood of using PNC. Further down the list, there are some relatively smaller yet contextually significant impacts on variables such as: Marriage Age Group 1621, Husband Occupation Service Holder, record Women Age groups, and Children Ever Born 3 or more children, among others. In general, the plot shows that the main behavioral and socioeconomic determinants of maternal PNC uptake in Bangladesh are place of delivery, husband’s occupation, residence, wealth status, and media exposure.

## 4. Discussion

**Fig 8:**
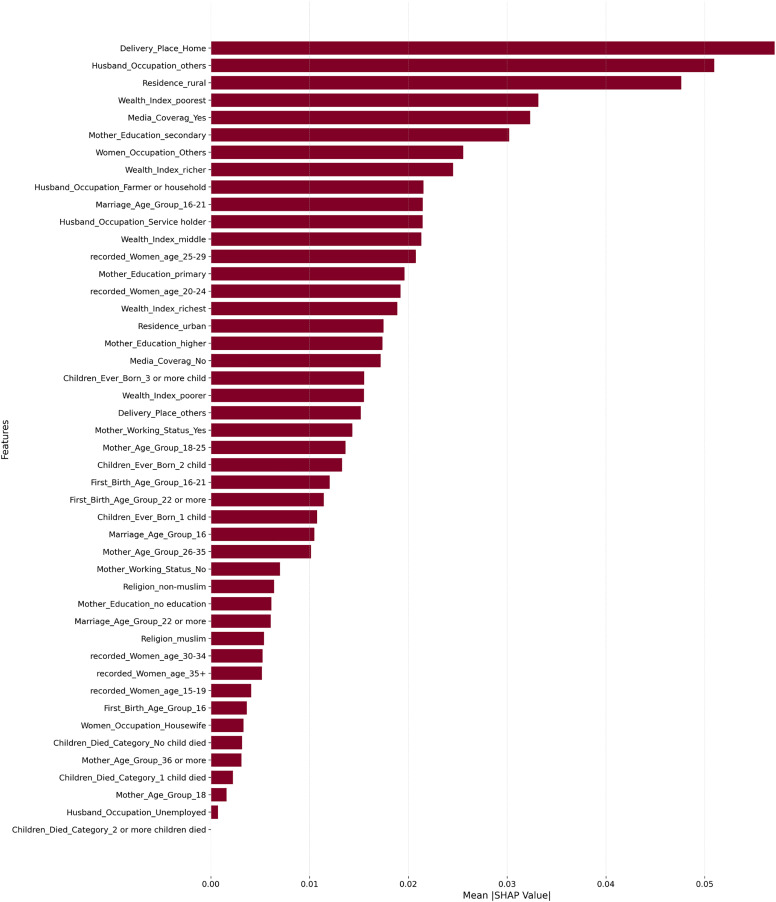
Explanation plot or shapley values plot for PNC.

The results of our machine learning model on postnatal care (PNC) utilization in Bangladesh offer an insight into the multivariate factors influencing service uptake in maternal health. SHAP value analysis provided a transparent rank of predictors, showing socioeconomic, demographic, geographic, and behavioral predictors, as shown in Fig 8. SHAP values were computed using the Random Forest model’s TreeSHAP implementation, with mean absolute SHAP values used to rank predictors by their overall contribution to model output. Rankings were stable across multiple random seeds.

Although logistic regression yielded broadly similar insights, ML added value by handling class imbalance, capturing nonlinear relationships, and ranking predictors through SHAP. This allowed us to uncover patterns that may be overlooked in traditional regression, while still maintaining interpretability.

Delivery Place Home was the strongest predictor of maternal PNC utilization, with the highest mean absolute SHAP value among all features, indicating that home births are associated with a considerable reduction in the probability of receiving PNC. This finding is consistent with numerous studies in Bangladesh that have identified facility-based delivery as a critical pathway to subsequent postnatal care uptake [28]. Husbands’ occupation emerged as the second most important predictor, with mothers whose husbands were in other or non-service occupations showing lower PNC utilization, reflecting that households where spouses occupy formal service positions tend to have greater resources and health awareness [29]. Residence in rural areas ranked third, further underscoring the persistent geographic disadvantage faced by rural mothers in accessing maternal health services.

The wealth index exhibited a clear gradient, with the richest quintile recording the highest PNC uptake (27.25%) compared to only 15.94% among the poorest. This socioeconomic disparity parallels findings from Bangladesh Demographic and Health Survey analyses showing persistent wealth-based inequalities in maternal health service utilization [30,31]. Maternal education further amplified service use: women with secondary schooling had 22.77% uptake compared to 20.41% among those without formal education, corroborating evidence from Bangladesh showing education as a dominant predictor of postnatal care utilization [32].

Geographic residence played a substantial role: urban mothers had 25.98% PNC uptake compared to 20.81% in rural areas, mirroring rural–urban inequities documented in Bangladesh’s maternal health services [33].

Media exposure appeared as one of the primary behavioral drivers in the SHAP analysis, with 23.12% of women who were media exposed having uptake, compared to 21.78% of women who were not media exposed. Though the bivariate chi-square test did not identify media exposure as statistically significant (p = 0.271), its high SHAP ranking indicates the Random Forest model’s ability to capture non-linear and interaction effects that univariate chi-square tests cannot. In particular, this media exposure can have a conditional effect; e.g., the effect of media exposure on PNC uptake can be exaggerated in rural or low-income women, a phenomenon that cannot be captured by chi-square but can be captured by ensemble models through feature interactions. It is this discrepancy that is a major strength of ML over traditional bivariate analysis: identifying complex, context-specific predictors. [34].

Other factors, such as religious affiliation and prior child mortality, had lower SHAP values, indicating smaller but contextually relevant influences on PNC decision-making, consistent with multilevel analyses of postnatal care determinants in Bangladesh [35].

From a methodological perspective, ensemble classifiers Random Forest (AUC = 0.9050), XGBoost (AUC = 0.9036), and CatBoost (AUC = 0.9028) outperformed traditional logistic regression (AUC = 0.8470), aligning with recent applications of machine learning in maternal health prediction [36,37]. SHAP analysis enhanced model interpretability, offering preliminary insights that may inform policy discussions, though caution is warranted in translating these findings into practice.

Strengthen rural maternity centers and emergency transport to increase institutional births and subsequent PNC uptake, as facility delivery remains a critical pathway to postnatal care [38]. Support girls in school through stipends and safe transportation, as education fosters autonomy and health literacy, with secondary education showing particularly strong associations with PNC utilization [32]. Leverage radio, television, and mobile platforms to disseminate maternal health messages, as media exposure consistently emerges as a significant predictor of service utilization [34]. Implement conditional cash transfers or subsidized maternal insurance targeting the poorest quintiles, addressing the substantial wealth-based disparities in PNC access [8]. Utilize community health workers and local leaders to deliver culturally sensitive interventions, particularly in rural areas where geographic barriers persist [33].

These results have significant policy implications and are directly correlated with SDG 3, which aims to achieve healthy populations and promote well-being for all people. In particular, minimizing wealth-based differences in PNC access helps achieve SDG 3.1 (reducing maternal mortality), whereas enhancing facility-based delivery and rural health facilities helps achieve SDG 3.8 (universal health coverage). Further supporting SDG 3.7 and SDG 4.1, respectively, are expanding media-based health communication, investing in female education, and emphasizing the necessity of cross-sectoral policy responses.

Longitudinal BDHS analyses can track PNC utilization trends and intervention impacts over time. Integrating spatial GIS data and real-time analytics will enable localized policy optimization. Further exploration of hybrid deep-learning models with SHAP interpretability may enhance precision while retaining transparency, building on emerging applications of explainable AI in maternal health [39,40]. These results indicate long-standing structural inequalities, including wealth-based differences, urban-rural inequalities, and disparities in education levels that determine PNC access beyond preference. To solve these determinants, the supply aspect of skilled provider availability should be improved alongside the demand aspect of increasing education and health communication.

## 5. Strengths and limitations

The paper contains several key strengths, among which the application of advanced machine learning models, such as Random Forest (AUC = 0.9050), XGBoost (AUC = 0.9036), and CatBoost (AUC = 0.9028), contributed to a high reliability of predictions of postnatal care (PNC) utilization based on the 2022 BDHS data. Analysis of SHAP values rendered the findings interpretable, identifying delivery place, husband’s occupation, rural residence, wealth index, and media coverage as the most significant predictors of maternal PNC utilization. Model performance on imbalanced data was further improved by applying ADASYN oversampling exclusively within training folds to prevent data leakage and preserve the integrity of model evaluation.

Limitations, however, include the fact that the data are cross-sectional, so causality cannot be established. Some models performed comparatively poorly in handling class imbalance, and the failure to capture data on healthcare quality and cultural aspects could be a shortcoming of the analysis. Another weakness is that the BDHS dataset failed to record the attendance at delivery or the presence of a skilled birth attendant, and also failed to adequately capture cultural norms that affect the decisions mothers make about their care. Our 80:20 random split may result in mothers from the same DHS clusters appearing in both the training and test sets, potentially inflating performance estimates. While cross-validation mitigates this risk, future work should use group-wise or nested splitting to fully account for the survey’s hierarchical structure. Future research should also incorporate calibration metrics and decision curve analysis to evaluate clinical utility alongside predictive accuracy.

## 6. Conclusion

In conclusion, the presented study shows that machine learning algorithms, specifically Random Forest, XGBoost, and CatBoost, can serve as a valuable addition to standard regression in predicting maternal postnatal care (PNC) utilization in Bangladesh based on the BDHS 2022 data. Although ML has shown incremental improvements in predictive performance over logistic regression, its greater value lies in its ability to identify complex and nonlinear patterns and transparently rank determinants through SHAP analysis. Delivery place home, husband’s occupation, rural residence, wealth index poorest, and media coverage were identified as the strongest predictors, followed by maternal education, women’s occupation, and age-related factors, including recorded women’s age and first birth age group.

These results support long-standing structural inequities in the use of maternal health services. Poor and rural women and those with low education levels continue to be the most susceptible to the under-use of PNC. To address these inequalities, interventions must operate at a multi-pronged level: encouraging facility-based deliveries, engaging skilled providers, investing in female secondary education, extending health media campaigns, and implementing financial support mechanisms aimed at the poorest households. Equitable access also demands culturally sensitive community outreach and mobilization of local health workers.

Meanwhile, there is reason for caution. The cross-sectional design of the BDHS data does not allow causal inference, and the absence of variables on skilled birth attendance or cultural practices is likely to oversimplify the role of delivery place in determining PNC uptake. Ethical considerations also play a significant role, and misusing predictive models to the disadvantage of vulnerable women through misclassification must be avoided.

Future studies need to incorporate longitudinal data, spatial analytics, and real-time health information to capture trends and local variations. Prediction can also be refined further using more advanced yet interpretable techniques, such as hybrid ensembles and deep learning models. Finally, ML is not an alternative to conventional epidemiological methods, but rather a decision-support tool that, when integrated with existing public health systems, can empower maternal healthcare systems and reduce disparities in postnatal care use in Bangladesh.
